# A Valuable Premium

**Published:** 1884-12

**Authors:** 


### A VALUABLE PREMIUM.

THE MEDICAL RECORD VISITING LIST FOR 1885 GIVEN AS A PREMIUM TO ALL OLD AND NEW SUBSCRIBERS.

To all old subscribers who will renew their subscriptions within the next thirty days, and to all new subscribers who will send us their subscriptions for the New Year, we will send this valuable visiting list, which costs $1.25, *free*. This is a rare inducement, and should be taken advantage of by every physician in the land.

JAS. P. HARRISON & Co., Publishers.

### ITEMS.

Dr. W. D. Bizzell has moved his office to 30½ Marietta street. He now occupies the office formerly occupied by Dr. John Thad Johnson.

We have received from H. Campbell & Co., 140 Nassau street, New York, Bartley's vest-pocket urinary test case. It contains a scientifically correct urinometer enclosed in a cloth bag to prevent breakage, a heavy glass test tube serving as a urinometer jar and test tube, a package of litmus test papers, a pipette for convenience in handling the urine, two vials to contain the test powders, and a spoon. It is designed for the use of physicians at the bedside of the patient, or for the office, by which a complete examination of the urine for clinical purposes can be made. It is neatly arranged in a morocco case to fit the vest-pocket, and is well worth the price asked for it—$2.00.

The November issue of the *Medical Chronicle*, of Manchester, England, edited by Jas. Niven, M. A., M. B., and W. J. Sinclair, and published under the direction of an able committee, has been received. It is a monthly cf large octavo size, 120 pages. It presents every evidence of being conducted by trained and accomplished editors. We gladly welcome it to our table, and wish for it the success it merits.

CONVERSATION IN THE OFFICE OF A LADY PRACTITIONER.—Lady Patient—"Doctor, I came to see if you would attend me in my coming trouble." "Certainly; when do you expect to be confined?" "On the 5th of January." The doctor turned to her obstetric list and found that she was previously engaged for that date, for she replied: "I am sorry, very sorry that I cannot: that is the day I expect to be confined myself."—*Canadian Practitioner.*

At the meeting of the New York Academy of Medicine, held October 16th, 1884, Dr. H. Marion Sims presented a life-like and striking bronze bust of his father, the late Dr. J. Marion Sims, which he had had cast in Paris, from the marble bust made some time since by the French sculptor, Du Bois.

The *Therapeutic Gazette*, published by Mr. George H. Davis, of Detroit, and edited by Dr. Wm. Brodie, will, after Dec. 31st, be published in Philadelphia, and will be edited by Drs. H. C. Wood and R. Meade Smith. The Gazette is doing a good work in its field, and has already achieved marked success.

A woman at Waterford, New York, recently gave birth to four children. They were all girls. Their combined weight at birth was twenty-eight pounds.

The contagious Pleuro-pneumonia of cattle now prevailing in several of the Western States is said to have been traced to a herd of cattle sent west from Baltimore. All the affected cattle west of Ohio are stated to be thoroughbred Jerseys.

Mr. Wm. H. Vanderbilt has recently given the College of Physicians and Surgeons of New York $500,000. According to the *Medical Record* this makes this College the richest in the United States.

G. P. Putnam's Sons will shortly publish, by arrangement with the Vienna publisher, a translation, prepared by Dr. Barney Sachs, with the authorization of the author, of Dr. Meynert's Treatise on Psychiatrie. The first part of the work, devoted to the anatomy and physiology of the brain, the publishers hope to have ready by the beginning of the new year. The work will be fully illustrated.

It is stated now that the price of muriate of cocaine is only thirty-five cents a grain, the first in New York sold for eighty-seven cents a grain.

We had a pleasant call a few days since from Capt. J. C. McMichael, editor of the Barnesville *Gazette*, the best weekly newspaper in Georgia.

To Dr. Koller, of Vienna, probably belongs the discovery of the use of cocaine in producing anæsthesia of the conjunctiva. He used a two per cent solution.

The Carroll county Medical Society, at a recent meeting, resolved to be more diligent in collecting their fees, and to practice for no persons who do not settle their bills once a year.

A number of prominent Georgia physicians have visited our city in the past few weeks. Among the number we met Drs. T. O. Powell, of Milledgeville, J. W. Bailey, of Gainesville, and J. Hogan, of Athens.

Dr. Henry F. Campbell, of Augusta, the able President of the American Medical Association, has recently been operated on for a cataract. The many friends of Dr. Campbell will rejoice to know that the operation was successful and that he is again at home.

We have received from Hon D. N. Speer, State Treasurer, his annual report to the Legislature. It shows the finances of the State were never in a better condition than at present, and stamps Col. Speer as a most excellent financier. The State never had a better treasurer.

Dr. Samuel M. Bemiss, professor of the theory and practice of medicine and of clin'cal medicine in the University of Louisiana, who during the epidemic of 1878 was the representative of the National Board of Health in that city, died suddenly on the evening of November 17th.

The Negro; His Origin and Destiny, is the title of a neatly printed pamphlet, which the author, Jackson T. Taylor, of this city, has left on our table. We have seen many complimentary notices of this work by leading newspapers of the South, and from what is said of it, regard it as eminently worthy of careful reading by every one.

The following extract from a letter received from Dr. Wm. Brodie of Detroit, editor of the *Therapeutic Gazette*, shows in what esteem THE JOURNAL is held in the West: "Your editorial on Battey, my old friend whom I knew when he was in our city years ago, is capital. I admire his genius; he deserves your encomiums. Your journal is a credit to Southern medical literature.

William Wood & Co. New York, announce that they will shortly begin the publication of a new work to be styled "A Reference Hand-book of the Medical Sciences." It will consist of six or eight royal octavo volumes of eight hundred pages each. The work will be edited by Albert H. Buck, M. D., and will contain contributions from over two hundred of the most prominent men in America and Europe.

The Baltimore *Trade Journal* remarks: "The *Southern World*, a journal of industry for the farm, home and workshop, published at Atlanta, Ga., is one of the most welcome of our exchanges. Besides devoting considerable space to the industries of the State, it pays close attention to Agricultural matters, to the care of poultry, to hygiene and the household generally. Literature is not neglected either, and the paper as a whole, is a credit to Southern journalism.

Meteorological Summary for October at this station as furnished by S. W. Beall, Seargent Signal Corps U. S. A., shows the following: Highest temperature 90.8°, date 3d; lowest temperature 33.6° date 24th; greatest daily range of temperature, 24 8°, date 16th; least daily range of temperature, 8.8° 28th; mean daily range of temperature, 18.1°; mean daily dew-point, 53 5°; mean daily relative humidity, 63.1; prevailing direction of wind, east.

We have received from Dr. J. N. McCormack, Secretary of the National Conference of the State Boards of Health, an invitation to attend the next meeting of the Congress which will be held on the 2d Wednesday in December in Washington, D. C. at the Ebbett House at 10 o'clock A. M. It is to be hoped that there will be a full attendance, and that the Conference may be able to influence the National Government to take the necessary precautions to prevent the importation of cholera to this country.

The North Carolina *Medical Journal*, one of our most valued exchanges thus mentions us:

The Atlanta MEDICAL AND SURGICAL JOURNAL, under its present management, is a very handsome periodical. Its mechanical work is excellent, and the editorial conduct of the JOURNAL is good, and steadily improving. Its pages have been adorned in the last few months with excellent engravings of some of the more prominent Georgians, among others we notice that of Dr. Henry F. Campbell and Dr. Robert Battey.

Messrs. J. H. Chambers & Co., St. Louis, announce that in December they will issue the first number of a new monthly periodical to be devoted to surgery. It will bear date January, 1885, and will appear regularly thereafter on the first of each month, being published simultaneously at St. Louis and London, Eng. Dr. Lewis S. Pilcher, of Brooklyn, formerly senior editor of the *Annals of Anatomy and Surgery*, will have editorial control of the new journal. He will have associated with him Mr. C. B. Keetley, F. R. C. S., of London. It is announced that each number of the journal will contain from eighty to one hundred pages of reading matter. The subscription price is $5.00 a year.

The well known house of Henry C. Lea's Son & Co., of Philadelphia, announce that they will in February next, issue the first volume of "The American System of Practical Medicine," edited by William Pepper, M.D., LL.D., Provost and Professor of the Theory and Practice of Medicine and of Clinical Medicine in the University of Pennsylvania, assisted by Louis Starr, M.D., Instructor in Diseases of Children at the University of Pennsylvania. The work will consist of five imperial octavo volumes containing one thousand pages each, with illustrations. We have been furnished with a list of authors and subjects of this work, and their well known ability warrants us in saying that the book will have what it deserves to have, a large circulation. It will be sold only by subscription at the following prices per volume: cloth, $5.00; leather, $6.00; half Russia, $7.00.

A man in Macon attempted suicide some weeks ago, and the *Telegraph and Messenger* came out the next morning with the following:

BATTLE OF THE DOC'S.

Who saved Christopher?
"I," said Stevens;
"With sulphate of atropia
I fought the morphia,
I saved Christopher."

Who saved Christopher?
"I," said Blackshear,
"With the sweet simplicity
Of induced electricity,
I saved Christopher."

Well, who saved Christopher?
"I," said Moore,
"I stood by his side
While the physic was tried,
I saved Christopher."

But what saved Christopher?
"The pump," said Mettauer
"The stomach pump's spout,
Turned the pizen all out,
The pump saved Christopher."

Mr. E. Duncan Sniffen, 3 Park Row, New York, the well known advertising agent, makes the following truthful remarks in the New York Tribune, October 4th, regarding newspaper advertising:

"The newspaper is so comprehensive in its scope, so universal in administering to the wants of all classes, and of every occupation; it brings, as it were, the financial and commercial markets of the world to our counting-rooms, so that it may be truly said that a good advertisement in a widely circulated newspaper is the best of all possible salesmen—one who never sleeps and is never weary; who goes after business early and late; who accosts the merchant in his store, the lawyer in his office, the student in his study, the cultivated woman at the family fireside; who can be in a thousand places at once, and address a million of people each day, saying only the best thing at the right time and in the best manner.

"Now this typical salesman talks only about his own business in his own interest, and if in a crowd, he must, in order to secure a hearing, be more conspicuous than his competitors, and at all times he must be as attractive as possible. The work involves intelligence, a good deal of ingenuity, and original and ready resource to make the stale matter of yesterday fresh and inviting to-day. This is the kind of newspaper advertising that it pays to do, and that we undertake to do." Advertisers should send for E. Duncan Sniffen's Advertiser's Reference Book, 1884, as it is full of valuable information about leading newspapers, their circulation, rates, etc.

It appears now that to Dr. Samuel R. Percy, of New York, should be given the honor of the discovery of Cocaine or as he called it "Erythroxyline." He writes to the *Medical Record* of November 15th as follows : "On November 4, 1857, I read before the New York Academy of Medicine an exhaustive paper upon the leaf of the plant *erythroxylon coca,* and stated that I was busy in its chemical investigation. On December 2, 1857, I exhibited to the Academy of Medicine one scruple of the alkaloid of the leaves to which I gave the name erythroxyline. At the same time I left with the librarian of the Academy this scruple of erythroxyline, a quantity of fluid extract and some solid extract, and also a fine sample of the leaves. I read before the Academy the method of preparing the alkaloid and a number of physiological experiments upon dogs. I then stated that the hydrochlorate of erythroxyline had a peculiar but not unpleasant benumbing and paralyzing effect upon the tongue, unlike that produced by aconitia and not so persistent. The librarian of the Academy has been unable to find the paper or alkaloid, but the fact of such a paper having been read, and such an exhibit having been shown, is recorded on the books of the Academy. I st ll hope to find the paper. Some three years after this, Niemann, in Germany, discovered the same alkaloid, and in his announcement called it 'cocaine.' Immediately upon this announcement I reiterated the priority of my discovery in Dr. Stephen Smith's Medical Times. I also personally saw Mr. George Wood, of Philadelphia, and he promised that in the next edition of the United States Dispensatory the subject should be fairly stated. He forgot it. The name cocaine is not a correct one, it is almost universally called cocoine, leading one to suppose that it is a product from the cocoa berry. With its correct name erythroxyline it cannot be mistaken. As an American, I claim the name given to it in America, and hope that every American surgeon will do the same."

GASTON'S OPERATION.—The following merited compliment to our worthy contributor is from an editorial in Gaillard's *Medical Journal*, for October. It will be remembered that we published a description of the operation in our September number:

"Gaston's Operation, as it will hereafter be called, is well described in the article contributed by its originator in this number of the *Journal*.

"Most well-informed physicians have been aware that, from a variety of causes, the duodenal or inferior extremity of the ductus communis choledochus becomes closed, producing a series of pathological phenomena too well known to require description. Dr. J. Marion Sims and others have endeavored by the operation of cholecystotomy or the establishing of a fistulous discharge of the bile externally, to correct the evils and remove the danger of a closure of the duodenal end of the common duct; but, as is well known, the operation has been attended with fatal results. But even had it for the time so far succeeded as to secure the establishment of the fistulous discharge desired, all who are familiar with the physiological problems presented must realize that such success would be only apparent or temporary, and not real or absolute, as the outward discharge of the bile would practically so destroy nutrition, or so impair it, as to render death, as a rule, inevitable. Indeed, the fear of such a result must have deterred Hughlings Jackson, Petit, Thudicum, Langenback, Tait and others from performing the operation of cholecystotomy, for they have often discussed its feasibility, and have had abundant opportunity for performing it. Sims, it is well known, really did perform the operation, and with his usual enthusiasm hoped for wonderful results. He did not remember, or apparently did not remember, that had he succeeded in saving the life of his familiar patient, death must have resulted finally from the diversion of the bile from the duodenum, with the consequent subversion of the digestive processes.

"It has occurred to Gaston, and only to Gaston, so far as is known, to establish for the bile a fistulous opening, not through the abdominal walls (cholecystotomy) with an external discharge, but through the walls of the sac and the neighboring intestine, the bile being thus delivered in the intestine and not externally. His method of accomplishing this ingenious, great, novel and original operation is carefully described in his paper, as published in this number of the *Journal*.

"Gaston has succeeded in his effort, and has created and contributed a new, bold, and life-saving operation. He is entitled to honor and distinction, and his brethren everywhere will fervently and cheerfully accord to him all that he so deserves and has so fairly and fully won."

Dr. A. W. Calhoun informs us just as we go to press that the Solution of Hydrochlorate of Cocaine begins to deteriorate after a short time. This fact should be noted and large quantities of the solution should not be made up.

Dr. R. O. Colter, after spending several weeks in the West, has returned and opened an office at No. 48 Marietta Street.

